# Periodontal Disease, Inflammatory Cytokines, and PGE_2_ in Pregnant Patients at Risk of Preterm Delivery: A Pilot Study

**DOI:** 10.1155/2018/7027683

**Published:** 2018-08-01

**Authors:** Catalina Latorre Uriza, Juliana Velosa-Porras, Nelly S. Roa, Stephani Margarita Quiñones Lara, Jaime Silva, Alvaro J. Ruiz, Francina Maria Escobar Arregoces

**Affiliations:** ^1^Centro de Investigaciones Odontológicas (CIO), Professor Graduate Program in Periodontics, Faculty of Dentistry, Pontificia Universidad Javeriana, Colombia; ^2^Centro de Investigaciones Odontológicas (CIO), Faculty of Dentistry, Pontificia Universidad Javeriana, Bogotá, Colombia; ^3^Hospital Universitario San Ignacio, Colombia; ^4^Department of Clinical Epidemiology and Biostatistics, Faculty of Medicine, Pontificia Universidad Javeriana, Colombia

## Abstract

Periodontal disease is an infection that, in pregnant women, can act as a risk factor for preterm delivery by increasing local and systemic inflammatory responses.* Objective*. To analyze the presence of periodontal disease, proinflammatory cytokines, and prostaglandin E_*2*_ (PGE_2_) in pregnant patients at high risk for preterm delivery.* Materials and Methods*. Pilot study for a case-control study. We included 46 pregnant patients (23 patients at risk of preterm delivery as cases and 23 patients without risk of preterm delivery as controls). We excluded patients who received periodontal treatment, antibiotics, or antimicrobials over the last 3 months as well as those with infections or diseases such as diabetes or hypercholesterolemia. The patients underwent a periodontal assessment, and their levels of cytokines (interleukin- [IL-] 2, IL-6, IL-10, and tumor necrosis factor- [TNF-] *α*) and prostaglandin E_2_ (PGE_2_) were quantified.* Results*. Patients with periodontal disease showed higher levels of cytokines (IL-2, IL-6, IL-10, and TNF-*α*) and PGE_*2*_. Patients at high risk for preterm birth showed higher IL levels compared with patients at low risk for preterm delivery. PGE_*2*_ increased with the severity of periodontal disease. PGE_*2*_ was higher in patients at low risk for preterm delivery, although this difference was not significant.* Conclusion*. Periodontal disease can increase the systemic inflammatory response as well as the levels of PGE_*2*_ and inflammatory cytokines in pregnant patients.

## 1. Introduction 

Pregnancy is a physiological process associated with adverse outcomes such as preterm birth (<37 weeks), low birth weight (<2500 grams), or very low birth weight (<1500 grams). The global incidence of preterm delivery is 9.6%, which is equivalent to 12.9 million premature infants. Currently, preterm delivery is the second most common cause of death in children younger than 5 years after pneumonia. Each year, 1 million premature infants die. In addition, preterm birth is associated with deterioration in growth, cognitive and visual alterations, and learning disabilities [1, 2].

The adverse outcomes of pregnancy are associated with elevated systemic inflammatory mediators and intrauterine infections. Current evidence suggests that preterm delivery is primarily due to ascending infections from the vagina or cervix or via hematogenous spread from nongenital sources.

Significant evidence supports an association between the spread of pathogenic bacteria associated with moderate/severe periodontitis and infections and extraoral inflammation. The virulence properties assigned to specific oral pathogenic bacteria (e.g.,* F. nucleatum, P. gingivalis, C. rectus, *among others) might be related to the adverse results of pregnancy, which might partially explain the biological plausibility of the associations among periodontal disease, periodontopathogenic bacteria, systemic inflammatory mediators, and adverse pregnancy outcomes [3].

Maternal periodontitis might represent a nongenital source of microorganisms that, because of their routine entry into the circulation system, have the potential to influence the health of the fetal-maternal pair. Periodontal infection and its pathogens generate virulence factors via the production of lipopolysaccharides (LPSs) and increased cytokines such as tumor necrosis factor- (TNF-) *α*, interleukin- (IL-) 1, IL2, and IL-6 as well as inflammatory mediators such as prostaglandin E2 (PGE_2_) [1, 4].

During pregnancy, changes in maternal physiology and metabolism occur; for example, peripheral vascular resistance decreases to compensate for the increases in renin and angiotensin. This effect is attributed to specific prostaglandins (PGE_2_ and PGI_2_).

In a normal pregnancy, these prostaglandins are synthesized in the fetal membranes: the decidua, the myometrium, and the placenta. The amnion and chorion primarily produce PGE_2_; the decidua synthesizes PGE_2_ and PGF_2_; and the myometrium secretes PGI_2_. The placenta produces a large amount of PGI_2_ that protects against thrombosis during low pressure in the intervillous space. The production of these substances is proportional to gestational age such that more PGs are present at the end of pregnancy than during the first trimester. Inhibition decreases over time. Prostaglandins also increase the number of myometrium receptors for oxytocin [5, 6].

At the end of a normal term pregnancy (more than 37 weeks) the levels of PGE_2_, TNF-*α*, and IL-1*β* are increased until reaching critical levels, which initiates uterine contractions and produces birth [7, 8]. However, during pregnancy and before the end of term, the proinflammatory immune response must be strictly regulated within the uterus to avoid the immune rejection of the fetus. A local increase in proinflammatory mediators can interrupt this delicate balance; therefore, an inflammatory response stimulated by a local infection (e.g., periodontal disease) under this mechanism would contribute to the premature rupture of membranes and uterine contraction, thereby triggering preterm delivery or spontaneous abortion [7, 9, 10].

Therefore, the objective of this work was to analyze the cytokines IL-2, IL-6, IL-10, and TNF-*α* and inflammatory mediators such as PGE_2_ in pregnant patients at risk of preterm delivery and their relationships with periodontal disease.

## 2. Materials and Methods

To achieve the proposed objective, a pilot case-control study was conducted.

Pregnant women with a gestational age greater than 20 weeks were considered for inclusion. Patients who received periodontal treatment, antibiotics, or antimicrobials over the last 3 months or who showed active infections or underlying diseases such as diabetes or hypercholesterolemia were excluded from this study.

We analyzed 46 pregnant patients (23 cases and 23 controls). The cases were pregnant patients considered at risk of preterm delivery; the controls were pregnant women not at risk. The evaluated exposure was periodontal disease as a local infection; inflammation and the systemic inflammatory response were analyzed based on the presence of cytokines such as IL-2, IL-6, IL-10, and TNF-*α* as well as inflammation mediators such as PGE_2_.

The risk diagnosis of preterm delivery was classified as follows: (1) threat of preterm birth (patients with uterine activity without cervical changes); (2) preterm labor during the initial phase (patients with regular uterine activity with a cervical change less than 4 cm); and (3) advanced phase preterm labor (patients with uterine activity with a cervical change greater than 4 cm). This classification was determined based on the Clinical Practice Guidelines of the Obstetrician-Gynecology Service of San Ignacio University Hospital and was performed by a trained gynecologist [8].

The patients in the case group were hospitalized at San Ignacio University Hospital and presented with the risk of preterm delivery in stages 1 and 2 (i.e., the threat of preterm labor or in the initial phase of preterm labor). Pregnant patients were included in the control group if they had received routine gynecological care on an outpatient basis and did not present with the risk of preterm delivery.

This research was classified as minimal risk in accordance with the regulations established by Resolution 8430, Ministry of Health of Colombia and CIOMS [11, 12]. The Research and Ethics Committees of the Faculties of Dentistry and Medicine at Pontificia Universidad Javeriana provided approval, and each patient was informed about the goals of the research, its scope, and possible benefits before entering the study. After their questions were answered and they showed full understanding of the process in which they would participate, they were asked to sign an informed consent document.

The patients who entered the study provided a blood sample, and two tubes were obtained: one tube to analyze the cholesterol, triglycerides, HDL, LDL, basal glycemia, and hematocrit levels and a second tube to quantify the levels of cytokines and PGE_2_. The second tube was centrifuged immediately at 10,000 rpm for 10 minutes at room temperature to obtain blood serum and frozen at -20°C until use. IL-2, IL-6, IL-10, and TNF-*α* were quantified using the BD CYTOMETRIC BEAD ARRAY (CBA) Human Th1/Th2 Cytokine Kit II (reference: 55181) following the manufacturer's instructions. Samples were acquired in the Hospital Universitario San Ignacio, using the FACSCanto II flow cytometer from Beckton Dickinson, and the data were analyzed using FCAP Array software.

PGE_2_ was quantified using the ELISA technique via the Human PGE_2_ kit reference KHL1701 (Invitrogen) following the manufacturer's instructions. The absorbance levels were recorded using a spectrophotometer at a wavelength of 412 nm, and the values obtained were transposed to the values given by the standard curve that comes with the kit and replaced in the formula suggested by the kit. Thus, the calculated results were given in picogram units per milliliter (pg/ml).

All of the patients underwent a periodontal evaluation after completing the periodontogram, during which a North Carolina periodontal probe was used. Only two people calibrated to perform periodontal examinations conducted this assessment. Periodontal disease was diagnosed based on the 1999 Armitage classification [13].

For data analysis, the demographic characteristics, the results of the periodontal evaluation, the cytokines, and the PGE_2_ were described using means, medians, ranges, standard deviations, and 95% confidence intervals. A bivariate analysis was performed in which the levels of the cytokines and PGE_2_ were compared between groups, and the periodontal diagnosis was examined using ANOVA after ensuring the normal distribution of the variable data (*α* < 0.05).

## 3. Results

The sample consisted of 46 patients who were divided into two groups based on the risk of preterm delivery. The case group consisted of 23 patients at risk of preterm delivery or high risk (HR) and the control group consisted of 23 patients not at risk or normal (N). The average age was 25.9 years (SD = 6.4 years), and the average gestational age was 29.4 weeks (SD = 4.3). The following periodontal diagnoses were determined: two patients were healthy (4.3%), 21 had gingivitis (45.7%), and 23 had chronic periodontitis (50%).

The mean gestational ages were 31 weeks for women at risk of preterm delivery and 28 weeks for women without risk of preterm delivery (p = 0.466). Both groups had an average age of 26 years.

## 4. Oral Clinical Characteristics

When analyzing the oral clinical variables with regard to the percentage of biofilm, number of teeth, number of teeth with chronic periodontitis, number of teeth with gingivitis, percentage of teeth with chronic periodontitis, and percentage of teeth with gingivitis, no significant group differences were found. The percentage of biofilm (62%) and the number of teeth with gingivitis (21.8) clinically was higher in the group at risk for preterm delivery, but this difference was not significant (Table 1).

## 5. Periodontal Diagnosis

The between-group analysis with regard to periodontal diagnosis chronic periodontitis (14 High Risk, 9 Normals), gingivitis (9 High Risk, 12 Normals), and healthy periodontal (0 High Risk, 2 Normals) revealed that more pregnant women in the group at risk of preterm delivery had more chronic periodontitis, and this group had no healthy periodontal patients.

## 6. Quantification of PGE_2_

**Figure 1:**
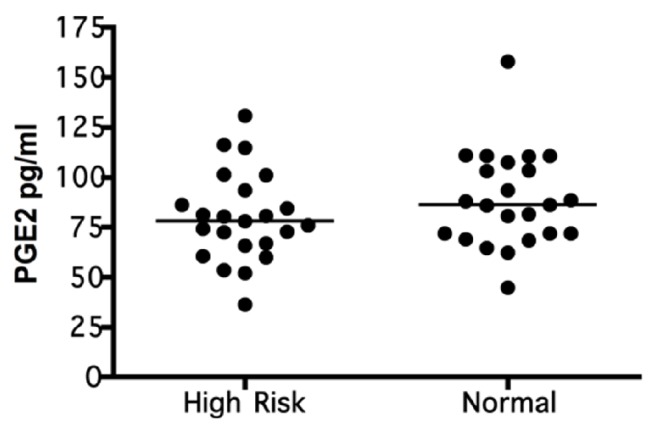
**PGE**_2_ **levels by risk.** Expression of PGE_2_ in case group patients at risk of preterm delivery (High Risk) and the control group not at risk (Normal), n=23 per group. The PGE_2_ levels was quantified by ELISA and compared between groups by a bivariate analysis. The data are represented by the median for each group.

With regard to the level of PGE_2_, an average of 84.4 pg/ml (SD = 23.2) was found, with a minimum of 36.2 pg/ml and a maximum of 157.8 pg/ml. According to the Kolmogorov-Smirnov test, this variable was normally distributed (p = 0.695), which enabled the later evaluations by group differences (with risk versus without risk) and periodontal diagnosis (healthy versus chronic gingivitis versus chronic periodontitis) (Figure 1).

The comparison of the averages of the two study groups revealed that women at risk for preterm delivery had PGE_2_ levels of 79.9 pg/ml (22.3), whereas those without risk showed PGE_2_ levels of 88.9 pg/ml (SD 23.8); however, this difference was not significant (p = 0.196).

**Figure 2:**
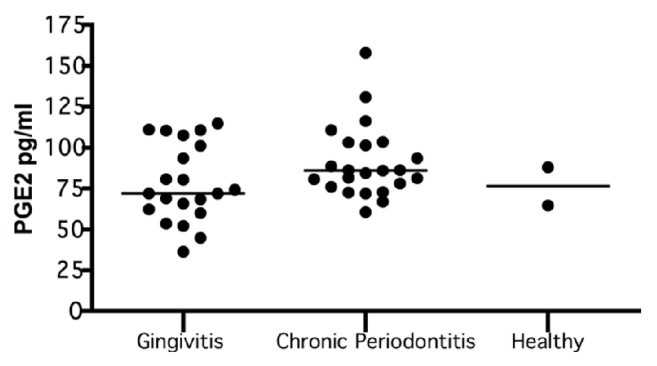
**PGE**_2_ **levels by periodontal diagnosis.** Expression of PGE_2_ in gingivitis (n=21), chronic periodontitis (n=23), and healthy (n=2). The PGE_2_ levels was quantified by ELISA and compared between groups by a bivariate analysis. The data are represented by the median for each group.

When PGE_2_ was evaluated with regard to periodontal diagnosis, the results revealed that the severity of the periodontal disease increased with the levels of PGE_2_. No significant differences were found with regard to periodontal diagnosis (p = 0.168), which might be due to the sample sizes of the different groups (Table 2; Figure 2).

The stratified analysis by group and periodontal diagnosis revealed the severity of periodontal disease increased with the level of PGE_2_, regardless of the group to which the woman belonged. The data also showed that these levels were the highest in the group of patients without risk of preterm birth but with chronic periodontitis. No significant differences were found based on group or periodontal diagnosis (p = 0.196; Table 3).

Subsequently, the cut-off point for the level of PGE_2_ was established based on normally distributed reference values (3-12 pg/l). All 46 women in this study, regardless of group or periodontal disease, had high (i.e., abnormal) values of this marker.

## 7. Quantification of Cytokines

**Figure 3:**
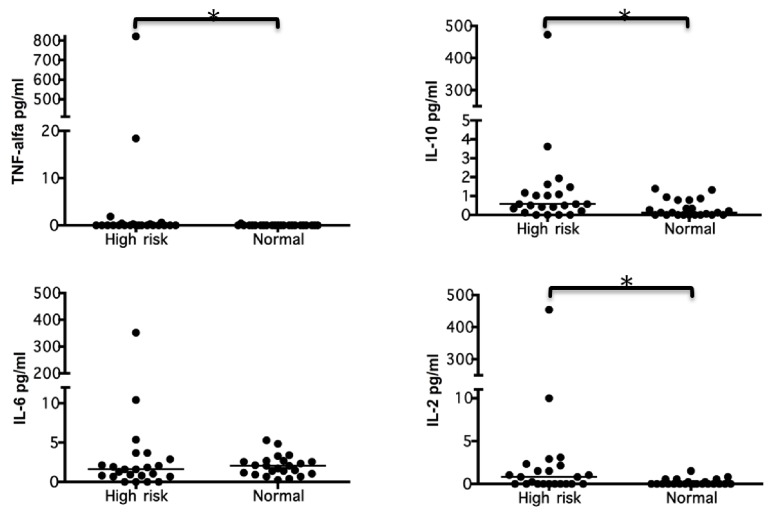
**The quantification of systemic cytokines in pregnant patients with **(High Risk)** and without the risk **(Normal)** of preterm birth.** TNF-*α*, IL-10, IL-6, and IL-2 were quantified using the CBA and by Flow Cytometer 50,000 events were acquired and analyzed using FCAP Array software. The concentrations obtained were expressed in pg/ml and compared between groups by a bivariate analysis. The data are represented by the median for each group (n=23 per group).

The patients at high risk of preterm birth had higher levels of cytokines (IL-2, IL-6, IL-10, and TNF-*α*) than those at low risk; significant differences were observed with regard to IL-2 (p-value 0.013), IL- 10 (p-value 0.012), and TNF-*α* (p-value 0.010) (Figure 3).

**Figure 4:**
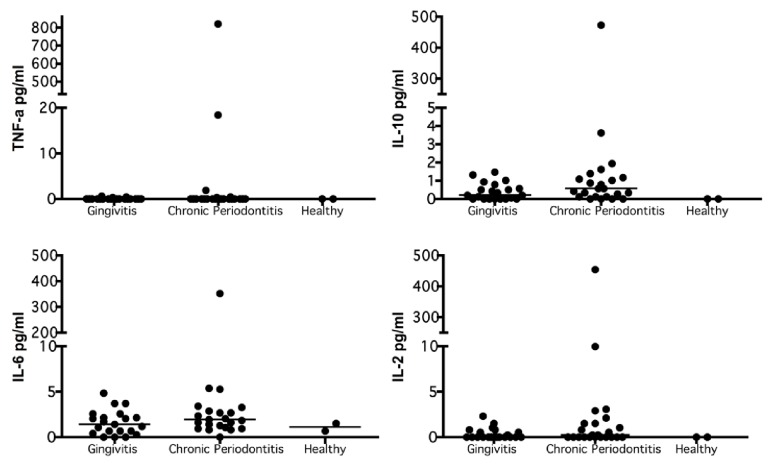
**Quantification of cytokines by periodontal disease.** Expression TNF-*α*, IL-10, IL-6, and IL-2 in gingivitis (n=21), chronic periodontitis (n=23), and healthy (n=2). Systemic cytokines were quantified using the CBA and by Flow Cytometer 50,000 events were acquired and analyzed using FCAP Array software. The concentrations obtained were expressed in pg/ml and compared between groups by a bivariate analysis. The data are represented by the median for each group.

The relationship between cytokines and the severity of periodontal disease revealed that, as the latter appears and increases in severity (from healthy to gingivitis to chronic periodontitis), the levels of the former increase. The highest values were found among patients with chronic periodontitis (Figure 4).

## 8. Discussion

Childbirth is a complex mechanism which has not been fully elucidated. Most, if not all, events that induce labor can be attributed to the action of the PGE_2_. In human and nonhuman primates, maternal plasma concentrations of PGE_2_ increase gradually during pregnancy and reach maximum levels at the end of pregnancy. Likewise, COX2 is expressed at low-to-undetectable ranges in the uterus during most of pregnancy, and it is highly regulated by proinflammatory cytokines and PGE_2_; COX2 catalyzes the production of prostaglandins in the amnion and plays a crucial physiological role in the initiation of labor by functioning as a potent activator of uterine contractions. PGE_2_ is positively regulated during preterm delivery; in turn, it is induced by an inflammatory response that promotes the contraction of the uterine smooth muscle [6, 14]. Periodontal disease, with its local and systemic bacterial load, can also trigger a systemic inflammatory response with increased inflammatory cytokines (TNF-*α*, IL-1, and IL-6) and inflammatory mediators (PGE_2_) to become a risk factor of preterm delivery.

The present investigation found that, as periodontal disease appeared and increased in its severity (from healthy to gingivitis to chronic periodontitis), the levels of cytokines (IL-2, IL-6, IL-10, and TNF-*α*) also increased, such that the highest values were found in pregnant patients with chronic periodontitis. These findings are consistent with the results of Zadeh 1999 [15] and Van Dyke et al. 2013 [16] who indicated that periodontal infection triggers an increase in IL-5, IL-6, IL-4, IL-10, IL-3, and TNF-*α* as a systemic inflammatory response.

In addition, investigations such as those by Greig et al. 1997 and Von Minckwitz et al. 2000 [17, 18] have indicated that the increased production of proinflammatory cytokines and inflammatory mediators in patients with periodontal disease, once released, can diffuse into the gingival crevicular fluid (GCF) or enter the bloodstream and reach the placenta-fetus interface. The cytokines IL-1, IL-6, and TNF-*α* stimulate the production of prostaglandins in the chorion and exacerbate cervical ripening and uterine contraction, which increase the risk of preterm labor. However, although the elevated serum and amniotic levels of these mediators are associated with several adverse outcomes of pregnancy, Carta et al. (2004), Deortbudak et al. (2005), and Goepfert et al. (2004) reported contradictory evidence by failing to find evidence that the increase of these mediators in gingival crevicular fluid, serum, or amniotic fluid in patients with periodontitis is associated with pregnancy complications [10, 19–21].

When analyzing PGE_2_, the present study showed that, as the severity of periodontal disease increases, the levels of PGE_2_ increased in pregnant women. These findings are similar to those reported by Konopka et al. (2003), who evaluated the relationships among periodontal disease, preterm birth, and low birth weight as well as the levels of PGE_2_ and IL-1*β* in the gingival crevicular fluid (FCG) and those in the blood serum of women with preterm labor and women who gave birth at term. They found that, in the presence of severe and generalized periodontitis, preterm delivery is 3.9 times more likely compared with women with a healthy periodontium. Among women with preterm delivery, significantly higher concentrations of PGE_2_ and IL-1*β* were found in the crevicular fluid [22].

Perunovik et al. (2016) evaluated the levels of four factors that trigger labor (PGE_2_, IL-1*β*, IL-6, and TNF-*α*) in the gingival crevicular fluid and serum samples among women with preterm birth and term delivery and correlate these levels with periodontal parameters. They found that women with preterm delivery exhibited significantly more periodontitis and showed an increase in the levels of IL-6 and PGE_2_ in the crevicular fluid compared with the term delivery group. Serum levels of PGE_2_ and TNF-*α* were positively correlated with probing depth and clinical insertion level (CAL) in women with preterm delivery [23].

The present investigation found that the levels of cytokines (IL-2, IL-6, IL-10, and TNF-*α*) were higher in patients at high risk for preterm delivery, supporting what is stated in the literature regarding the role that inflammatory cytokines play in the mechanisms that initiate labor. Similarly, Arntzen et al. (1998) [24] quantified the levels of TNF-*α*, IL-1, IL-6, and IL-8 in the amniotic fluid of women with preterm and full-term labor and found an association between increases in TNF-*α*, IL-1, and IL-6 levels and preterm delivery, suggesting that IL-1 plays a dominant role in the presence of chorioamnionitis, whereas IL-6 seems to be more important during preterm idiopathic delivery.

Our results might contribute to the biological plausibility of the relationship between periodontal infection and the risk of preterm delivery and justify what Amare Teshome et al. (2016) reported in a systematic literature review regarding the effect of periodontal disease on preterm delivery and low birth weight, which noted that periodontal disease increased the risk of preterm birth and low birth weight in 9 of the 10 articles analyzed, with an odds ratio (OR) that ranged from 2.04 to 4.19. Only one article did not report this association [25].

Likewise, Corbella et al. (2016) evaluated periodontal disease as an independent risk factor for the adverse outcomes of pregnancy. To accomplish this goal, they performed a meta-analysis to calculate the relative risk (RR) for each pregnancy outcome. The calculated RR of periodontitis was 1.61 for preterm birth assessed across 16 studies (p<0.001); 1.65 for low birth weight assessed across 10 studies (p<0.001); and 3.44 for low preterm birth weight assessed across four studies. They concluded that an association exists, albeit low, between periodontitis and adverse pregnancy outcomes [26].

Scientific evidence has shown some probable mechanisms of the relationship between periodontal disease and adverse outcomes of pregnancy. Unfortunately, the largest multisite randomized trials have failed to show a reduction in preterm birth even when the gold standard of treatment, deep scaling, and root planning was employed. Different meta-analyses show that the randomized clinical trials investigating the effect of periodontal treatment on adverse outcomes are of low quality because of high risk of bias, mainly due to the lack of blinding and to the heterogeneity of the trials [27, 28].

The results of this investigation could not determine whether the differential change of the patient's baseline in inflammatory cytokines and PGE2 is an absolute risk factor for preterm birth or if there is a real value beyond the risk of preterm delivery. Many women at risk of preterm labor, in fact, carry it to term. It would be interesting to include in future research only women with actual premature births and to compare them with term delivery controls.

Given the scientific evidence that increasingly supports the role of the systemic inflammatory response generated by remote infections (e.g., periodontal disease) regarding preterm delivery, it is worthwhile and justified to continue researching interdisciplinary groups involving both doctors and dentists to expand the sample and control the possible confounds in such a way that it is possible to visualize the true risk that a pregnant woman with a chronic infection such as periodontal disease might carry with regard to her pregnancy. In addition to this, periodontal disease and preterm delivery are two health problems of interest for public health; for this reason, if periodontal disease can be treated, dentists would be contributing to decrease the risk for preterm delivery.

## 9. Conclusions 

In pregnant women as the severity of the periodontal disease increases, PGE_2_ values increase. We observe that, in patients with chronic periodontitis, both those at high and low risk of preterm delivery had higher levels of PGE_2_; and patients at high risk for preterm labor and those with periodontitis had higher levels of systemic relevant cytokines that participate in both diseases. For this reason to have periodontal disease can favor the outcome of preterm delivery.

## Figures and Tables

**Table 1:** Oral clinical characteristics by group.

	**GROUP**		**p-value**
	**Risk**	**No risk**	
%** Biofilm**	62	45.8	0.519
**# Teeth**	28	27.9	0.249
**# Teeth with periodontitis**	2.5	2.0	0.642
**# Teeth with gingivitis**	21.8	17.4	0.050

**Table 2:** PGE_*2*_ levels by periodontal diagnosis.

**Periodontal diagnosis**	**Average PGE**_**2**_ ** (pg/ml)**	**SD**
**Healthy (n = 2)**	76.31	16.37
**Gingivitis (n = 21)**	78.11	23.76
**Chronic Periodontitis (n = 23)**	90.92	22.21

**Table 3:** PGE_2_ levels by risk of preterm delivery and periodontal diagnosis.

**Group**	**Periodontal diagnosis**	**Average PGE**_**2**_ ** (pg/ml)**	**SD**
**High risk**	Chronic gingivitis (n = 9)	70.86	24.83
	Chronic periodontitis (n = 14)	85.83	19.24

**Low risk**	Healthy (n = 2)	76.31	16.37
	Chronic gingivitis (n = 12)	83.55	22.42
	Chronic periodontitis (n = 9)	98.83	25.29

## Data Availability

The data used to support the findings of this study are available from the corresponding author upon request.
